# Nurse leader perspectives of organisational capacity for higher education: implications for nursing education policy

**DOI:** 10.1186/s12912-025-03312-5

**Published:** 2025-06-04

**Authors:** Patricia Y. Mudzi, Sfiso Mabizela, Judith Bruce

**Affiliations:** 1 Department of Nursing Education, School of Therapeutic Sciences, University of the Witwatersrand, 7 York Road, Johannesburg, South Africa; 2 Centre for Health Science Education, Faculty of Health Sciences, University of the Witwatersrand, Johannesburg, South Africa; 3 School of Therapeutic Sciences, Faculty of Health Sciences, University of the Witwatersrand, 7 York Road, Johannesburg, South Africa

**Keywords:** Capacity, Higher education, Nursing college, Organisational development, Policy

## Abstract

**Background:**

The location of nursing education in higher education is a global change initiative that intends to achieve an improved, highly qualified nursing workforce to enhance professional status in line with other health professions. Organisational capacity has become an emerging research interest and a critical success factor in change initiatives as nursing colleges strive to align with international trends and national accreditation standards. The aim of the study was to determine nurse leaders’ perspectives on the capacity of public nursing colleges for programme accreditation against higher education criteria and standards.

**Methods:**

A descriptive cross-sectional survey design was used. Data were collected from 88 nurse leaders in urban, rural, and mixed provincial nursing colleges across three South African provinces using the Higher Education Diagnostic Indicator of Readiness and Capacity to Change scale. Descriptive statistics, confirmatory factor analysis, and inter-item reliability were applied in data analysis and instrument testing.

**Results:**

Organisational diagnostics revealed weaknesses in human resources, technology, infrastructure, and research capacity amid well-grounded strategies for student recruitment and teaching and learning, and appropriate staff qualifications and experience. Staff shortages, research limitations, inadequate infrastructure, including outdated facilities and limited information technology resources were prevalent, falling below higher education standards.

**Conclusion:**

Diagnosing organisational capacity for change provides an empirical basis for a deeper understanding of the status of nursing colleges as functional higher education organisations. The perspectives of nurse leaders highlight critical areas to be addressed, which include key policy pillars for staffing, infrastructure resources, and ongoing professional development. Strengthening policy support for nursing college education is crucial for producing highly skilled nurses capable of meeting the evolving demands of modern healthcare systems.

**Clinical trial number:**

Not applicable.

## Introduction

There is growing universal consensus on nursing education being part of the higher education landscape. Nursing’s location in higher education intends to achieve an improved, highly qualified nursing workforce and to enhance professional status in line with other health professions [1]. Emphasised by the World Health Organisation [2], appropriately qualified nurses, on par with allied health professionals, maximise the contributions and roles of nurses in interprofessional teams. At the epicentre is the capacity of nursing education institutions to prepare nurses for these roles in a range of healthcare systems.

Over time, the transitioning of nursing education to higher education has been variously paced- it is ongoing and incomplete in some countries, particularly in Europe and Africa [3, 4], and in others, such as the United States of America and Australia, transitioning to higher education is complete [5]. In all these countries, change in nursing education is informed by global directives, policy or legislation [4]. Nursing education policy is essential to achieve the goals of educating nurses in a higher education context and to ensure alignment between higher education regulations and health service demands [6, 7]. However, successful transitioning requires more than a policy decision to become part of higher education. Research highlights organisational capacity and readiness as a critical factor for successful change initiatives in organisations [8, 9] to fulfil the legal requirements of a country’s education system. Examining organisational capacity for change in an educational context provides evidence-based content for the review and development of nursing education policy.

## Background

Organisational capacity for change has become an emerging area of research interest over the past decade, shifting the focus from how to prepare organisations to how to support them [8]. In this study, “capacity” denotes the capabilities, resources, and infrastructure available for an organisation i.e. a nursing college to meet the accreditation standards for higher education and to fulfil their mandate to produce qualified nurses and “change” refers to the integration of public nursing colleges into the higher education system. The capacity of nursing colleges to relocate to and function in higher education hinges on several factors: the ability to adapt to the social, technological, economic, regulatory, and political aspects inherent in becoming a nursing education institution in higher education [10]. Adapting to these factors is critical for institutions making the change, as they must align with the broader demands and expectations of higher education systems [7].

In most European countries, the Bologna Declaration has yielded positive outcomes for nursing education, generating consensus on nursing policy to harmonise and improve the qualifications of the nursing workforce [11]. Successful harmonisation of nursing education can be seen in Sweden and Finland, where there is a unified higher education framework that promotes quality and student mobility [12]. In South Africa, the push for universal health coverage and the Department of Higher Education and Training (DHET) policy imperatives have necessitated the repositioning of nursing colleges [6]. All nursing education institutions are required to comply with the accreditation standards of the Council on Higher Education (CHE) and the South African Nursing Council. Although accredited to offer higher education nursing programmes, nursing colleges must be formally recognised as a distinct institutional type under the amended Higher Education Act of 1997 [13]. Lacking formal recognition restricts the profession’s capacity to maximise the benefits of higher education. In Europe, the Bologna Declaration helped achieve policy consensus and unification; in South Africa, inadvertent policy gaps in institutional recognition and accreditation may hamper consensus and higher education status.

The transition of South African nursing colleges from health to higher education highlights the growing need for research offering context-specific insights and evidence to guide and review nursing education policy. The National Policy on Nursing Education and Training [14] is silent on accreditation matters and conditions for colleges to achieve higher education status. The rarity of nurse leader involvement in policy decisions and ineffective leadership skills [15] compound weaknesses in nursing policy.

### Organisational development framework

The Organisational Development (OD) framework by Cummings and Worley [16, 17] was selected as it examines the critical components organisations should have to navigate change successfully. The OD framework offers a valuable tool for investigating these components with particular attention to the general environment for programme accreditation standards and nursing education policies, which significantly impact the overall effectiveness of nursing colleges.

**Fig. 1:**
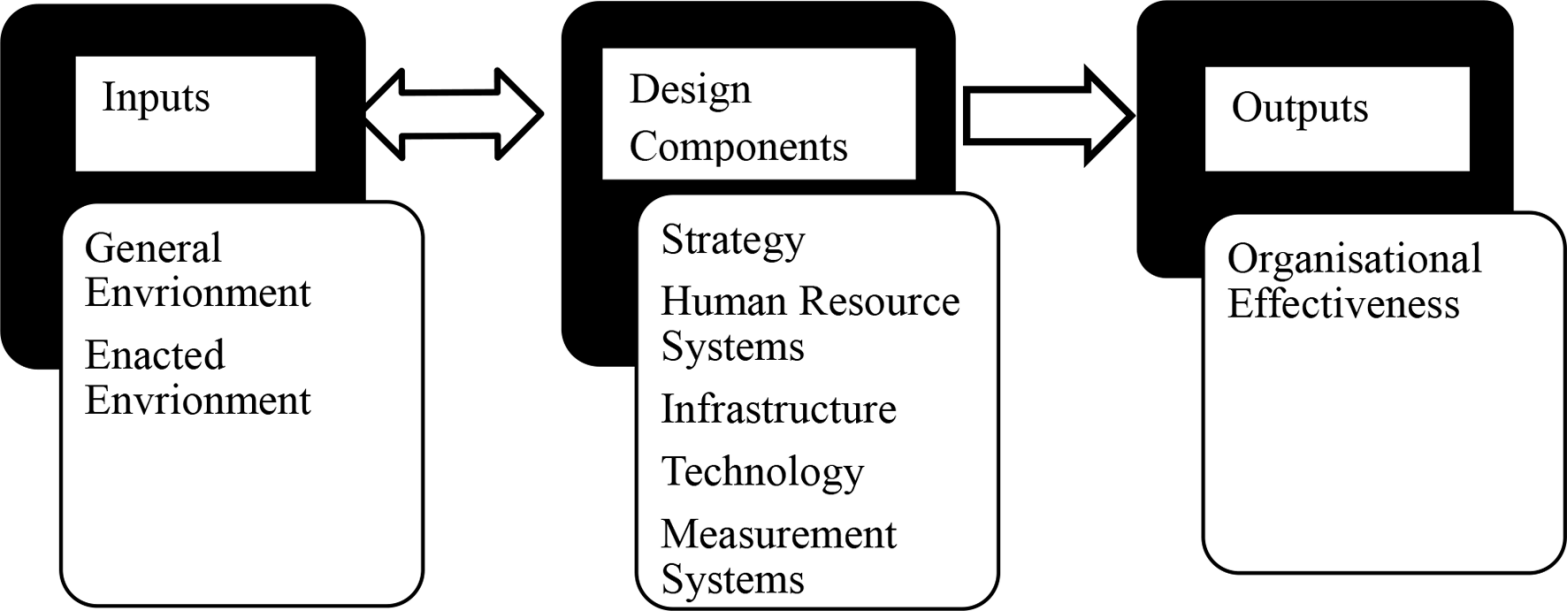
Adapted diagnosing activity at the organisational level [16]

This research is part of a study on nursing colleges’ readiness for higher education using an OD framework. The first phase, Entering and Contracting, identified gaps in transitional arrangements, governance, and policy development [18]. This paper addresses phase two, the Diagnosing, assessing organisational capacity, examining relevant policies and processes, and identifying areas for future development through insights from nurse leaders. The Diagnosing phase (Fig. 1) enables participants to compare structures (resources) with accreditation standards with the emergence of strengths and weaknesses as a corollary to understanding the capacity of nursing colleges to achieve the desired output: organisational effectiveness. An organisation’s overall effectiveness is determined partly, by the extent to which there is alignment between the organisation and its environment (inputs) and between inputs, design components, and outputs, and among the subsystems of the change process [16]. Through an organisational development lens, this study intended to provide diagnostic indicators of nursing college capacity to meet higher education accreditation criteria.

## Methods

### Study design

A descriptive cross-sectional survey design was used to collect and document key characteristics at a single point in time without manipulating study variables. The Diagnosing phase of the OD framework was applied. This phase, with its focus on generating diagnostic indicators, was particularly relevant for assessing how current practices within nursing colleges align with higher education policy.

### Sample and setting

Participants were recruited from public nursing colleges and directorates in Provincial Departments of Health (PDoH). Total population sampling was used to obtain a sample of 88 nurse leaders who held leadership or management positions in a provincial nursing college or directorate. The sample comprised nursing college Principals (*n* = 3), nursing college Campus and sub-campus Heads (*n* = 15), Heads of Department within nursing colleges (*n* = 25), Acting Heads of Department (*n* = 42), and one of three Nursing Education and Training Directors (*n* = 3) from the PDoH. Total population sampling was used since key specialised participants suited to nursing education leadership were required, reducing the risk of selection bias common in random samples.

The study was conducted in three South African provinces: urban, rural, and mixed urban-rural. Urban provinces have high population density and extensive infrastructure, while rural provinces feature lower density and limited services, often comprising small towns and villages. Mixed provinces include densely populated areas with developed infrastructure alongside sparsely populated rural communities engaged in agriculture. Only accredited nursing colleges offering higher education programmes were included.

### Data collection

Data collection procedures varied by province based on internet connectivity and the availability of nurse leaders. In the mixed urban and rural province, the survey was completed on a computing device and returned via email. In the remaining provinces, the survey was administered in person, in a paper-based format, in designated nursing college spaces, and handed to the researcher upon completion. On average, paper-based surveys took 35 min to complete, with no missing data. Data collection occurred intermittently over 16 weeks, from November 1, 2022, to June 30, 2023.

### Instrument

The self-administered Higher Education Diagnostic Indicators of Readiness and Capacity to Change (HEDIRCC) instrument was used to collect scaled responses from nurse leaders. The scale items were derived from the CHE programme accreditation criteria and standards, which all nursing education institutions must meet to offer nursing programmes [19]. The 49-item instrument consists of seven main criteria with standards (items) relevant to pre-registration programmes: student recruitment, admission, and selection (eight items); academic staff qualifications and experience (seven items); academic and support staff compliment (nine items); teaching and learning strategy (seven items); assessment policies and procedures (five items); infrastructure and library resources (seven items), and programme administrative services (six items).

The items measuring accreditation standards were scored on a 3-point Likert scale as follows: “Standard met” indicates that the minimum requirement for an accreditation standard within a specific criterion was satisfied or attained. If the minimum requirement was incomplete or partially attained, the standard was considered “partially met.” If the minimum requirement was not achieved or absent, the rating was “standard not met.” “Not applicable” meant that certain standards may not be relevant for a specific nursing programme.

The survey used in this study aligns with key elements of the Cummings and Worley (2015) Organizational Development Framework, specifically the Inputs, Design Components, and Outputs (Fig. 1). Under Inputs, items related to legislation, student recruitment, and equity reflect how colleges respond to their external environment. The Design Components are addressed through questions on staff qualifications, teaching strategies, infrastructure, and information systems, highlighting human resource systems, technology, and measurement practices. Finally, the Outputs dimension is reflected in items assessing student support systems, performance monitoring, and certification integrity, indicating overall organisational effectiveness. This alignment ensures the survey serves as a structured tool for assessing institutional readiness and capacity.

### Ethical considerations

Ethical approval was obtained from the University of Witwatersrand Human Research Ethics Committee (Certificate number: M220538). Participants received an information sheet and provided written informed consent, with the option to withdraw anytime. Nursing college and nurse leader names were coded to ensure anonymity and confidentiality; participant information was stored on a password-protected computer for researcher access only.

### Data analysis

Survey data were coded and captured in Microsoft Excel before being imported into SPSS 23 (IBM SPSS Statistical for Windows, Version 23.0, NY IBM Corp.). Confirmatory Factor Analysis examined how well the observed variables relate to the proposed factors (Table 1). This was preceded by assessing the suitability of scale items using the Kaiser-Meyer-Olkin (KMO) and Bartlett’s Test of Sphericity. The significance level was set at *p* < 0.05 for Bartlett’s Test of Sphericity, and the KMO value > 0.5. Descriptive statistics were used to determine frequencies for categorical data, and median and interquartile range (IQR) for continuous non-normal data.

## Results

### Demographic characteristics

The response rate was 85.2%, yielding a sample size of 75. Most nurse leaders (90.7%) were female based in an urban province (73.3%); 61.3% held a higher degree with more than 10 years’ experience in nursing education; 46.7% of nurse leaders were in temporary (acting) positions. Beginning the survey, participants had to indicate which nursing programmes had met the CHE accreditation standards. The three-year Diploma in Nursing was reported as meeting the standards by 80% of nurse leaders.

### Validity and reliability

Construct validity for the HEDIRCC showed that the criteria were intercorrelated; the p-value was set at *p* < 0.001. Inter-item reliability was calculated, yielding Cronbach Alpha values for the main criteria as follows: Student recruitment, admission and selection (0.633), Academic staff qualifications and experience (0.516), Academic and support staff compliment (0.887), Teaching and learning strategy (0.942), Assessment policies and procedures (0.867), Infrastructure and library resources (0.838) and Programme administrative services (0.906).

### The HEDIRCC confirmatory factor analysis results

The Confirmatory Factor Analysis results are illustrated in Table 1 above.

**Table 1:** HEDIRCC confirmatory factor analysis results

Criteria	Median Score (IQR)	Average Factor Loading	Average Alpha	KMO	Bartlett test
Student recruitment, admission, and selection	13 (11–15)	0.472	0.705	0.555	< 0.001
Academic staff qualifications and experience	11 (9–13)	0.461	0.604	0.615	< 0.001
Academic and support staff compliment	12(9–14)	0.543	0.779	0.712	< 0.001
Teaching and learning strategy	12 (9–14)	0.510	0.698	0.691	< 0.001
Assessment policies and procedures	8 (7–9)	0.469	0.575	0.560	< 0.001
Infrastructure and library resources	10 (8–12)	0.650	0.836	0.809	< 0.001
Programme administrative services	9(6–11)	0.596	0.762	0.719	< 0.001

### Student recruitment, admission, and selection

Most nurse leaders (63%) indicated that nursing colleges have marketing materials that present detailed programme information to new recruits. Nursing colleges are compliant with legislation that regulates student admission according to 84% of nurse leaders, including high school certification and age exemption rules (81.3%) and 61.3% respectively. Gaps were found in setting 5-year student equity targets, with 46.7% indicating this standard being met.

### Staff qualifications and experience

Whilst nursing college faculty (70.7%) reportedly meet the academic qualifications standard for higher education, and the teaching and professional experience standards (88% and 78.7% respectively), they do not possess the requisite qualifications to teach postgraduate programmes (42.7%). Half (50.7%) indicated that nursing colleges meet the standard for providing regular faculty development opportunities but fall short of the standard around the resources and external agencies for faculty development (45.3%).

### Academic and support staff compliment

This standard assesses faculty seniority and staff-student ratios needed for effective programme delivery. Most nurse leaders (57.3%) reported unmet or partially met standards for staff-student ratios and student numbers. Key gaps included full-time faculty ratios for teaching and learning (33.3%), research (22.7%), and diverse modes of teaching delivery (44%). Fewer nurse leaders rated administrative (48%) and technical staff (41.3) as adequately qualified to support nursing programmes compared to library staff (58%).

### Teaching and learning strategy

This standard focuses on promoting student learning and strategies to monitor progress, evaluate impact, and effect improvement and success. Most nurse leaders (80%) indicated that nursing colleges have a teaching and learning strategy appropriate for face-to-face instruction but not for online learning (13.3%). Other teaching and learning strategies that reportedly meet higher education standards have a programme implementation plan (74.7%), and ways to monitor student progress (73.3) and evaluate programme impact (64%);48% indicated that development opportunities to upgrade teaching methods meet the required standard.

### Assessment policies and procedures

Appropriate policies for assessment, moderation, monitoring student progress, and valid and reliable assessment practices are required for the different modes of nursing programme delivery. The majority (80%), pointed to well-established internal assessment policies and adequate academic peers, with 57.3% meeting the standard for moderating assessments. While most nurse leaders (66.7%) concurred that nursing college faculty have the requisite assessment experience, the standard for ongoing faculty development in assessment was either not met or partially met (54.7%).

### Infrastructure and library resources

According to 52% of nurse leaders, the standards related to physical facilities (simulation and computer laboratories) were either not met or partially met, while classrooms were considered sufficient by 68%. More than two-thirds (68%) considered information technology (IT) infrastructure as either not meeting or partially meeting the standard. Although nursing college libraries reportedly meet the accessibility standard (65.3%), fewer nurse leaders considered them compliant in having sufficient resources (45.3%) and providing research support (21.3%).

### Programme administrative services

This standard ensures that higher education programmes have effective administrative processes and policies to manage programme information and ensure the integrity of certification. Nurse leaders’ perspectives point to weaknesses in managing student admission and performance data and entering data into the National Learner Records Database. These standards were reported as either not met or partially met by 49.3% and 64% of nurse leaders, respectively. Despite shortfalls in data management, nursing colleges have systems to monitor student performance (60%), to identify students at risk (58.7%), and to refer students for appropriate support (62.7%).

## Discussion

This study uncovered considerable gaps in the capacity of nursing colleges to meet accreditation standards in higher education. The disconnect between policy directives and policy implementation leaves nursing colleges underprepared and insufficiently capacitated for change, emphasising the need for comprehensive, actionable strategies to bridge this gap. Nurse leaders viewed student recruitment, admission, and selection as meeting higher education standards. This positive view emanates from nursing colleges experience and a culture of adherence to national admissions policies to select students with the prescribed requirements. This approach contributes to nursing college compliance with accreditation criteria and effective higher education functioning [20]. Inadequacies in equity plans are an important diagnostic indicator, as nursing colleges risk flouting national laws and equity policies. Given South Africa’s history of racial discrimination and separate development, higher education policy demands improved access and success for students, specifically those whose racial classification, gender, or disability status had previously disadvantaged them [21].

There are shortcomings in the full-time faculty ratios for effective teaching and learning, for diversifying teaching delivery, and for research. Inadequate staffing levels present significant obstacles for nursing schools in South Africa and beyond [22]. Addressing this requires an analysis of staffing and faculty development policies and the conditions that restrict nursing colleges from attracting and retaining qualified faculty [23]. Not having sufficient research faculty and lacking the qualifications to teach in postgraduate programmes is a double-edged sword - this requires policy clarity about the type of organisation nursing colleges are to become in higher education [24], and their role in research and science development. Knowledge translation and evidence-informed policymaking are crucial in transitioning as they ensure that research advances academic knowledge and directly informs and improves healthcare practices [25]. These aspects of programme accreditation contribute to and validate nurse education quality and graduate outcomes that produce knowledgeable and skilled nurses for optimal healthcare provision [26].

The lack of resources for ongoing professional development in teaching methods and student assessment is a pervasive weakness across four accreditation criteria, compounded by a lack of contracted external expertise. Faculty members who continue using traditional teaching approaches do not fully address diverse student needs, and their reluctance to embrace online learning was found to affect student engagement and learning outcomes [27]. Despite having assessment policies in place, nursing colleges struggle with staff development in a rapidly evolving assessment environment [28]. This is particularly alarming given the crucial role of learning assessments in shaping student progress and academic performance in higher education.

Difficulties in adopting modern teaching methods and online learning are compounded by IT and physical infrastructure deficiencies and limited library resources. Persistent weaknesses in infrastructure, learning technologies, and human resources, as shown in this and other studies over the past 10 years [29], require policy alignment and policy guidelines on funding. Aligning infrastructure policies with the CHE accreditation requirements and sufficient funding would enable the necessary upgrades to physical and technological resources. National funding policies, specifically the equitable share funding model controlled by provincial health departments, present significant weaknesses [30]. This model lacks the specificity (i.e. not tied to specific conditions) required to adequately support the nursing colleges as they transition to higher education.

Amid a growing importance of online learning and digital health technologies, it is essential for nursing schools, specifically in sub-Saharan Africa, to be adequately resourced [31], concerning IT infrastructure, reliable internet connectivity, and qualified IT staff. Governments have the responsibility, through their ministries of health and higher education, to invest in nurse education and provide funding support on par with university nursing schools. This includes creating opportunities for nursing colleges to access and grow through earmarked grants using customised performance indicators. The Global Alliance for leadership in Nursing Education and Science (GANES) has developed a global framework for advancing and harmonising nursing education, globally - a key indicator is the availability of adequate resources for teaching and learning and appropriately qualified staff [10]. As social, cultural, and economic disparities exist between countries, some will continue to experience resource limitations in their quest for higher education status [10].

### Limitations

Data collection methods were administered using a combination of online and face-to-face approaches across the three provinces. In areas with poor internet access, face-to-face methods were prioritised to ensure participation and avoid incomplete data. The selection of geographically diverse provinces may have improved representativity; however, results are generalisable only to nursing colleges with similar characteristics. Additionally, the sample size of 75 participants was insufficient for a full-scale construct validation, and thus the findings from this study should be interpreted as preliminary. While the exploratory analysis of inter-item correlations provides early insights into the instrument’s performance, further validation with a larger sample is necessary to establish its robustness and generalisability. The two subscales (Student recruitment, admission and selection and Academic staff qualifications and experience) had low reliability values due to the small sample size, therefore these subscales may require further refinement in future iterations of the instrument.

## Conclusion

The activity of Diagnosing organisational capacity for change provides an empirical basis for a deeper understanding of the status of nursing colleges as functional higher education organisations. The perspectives of nurse leaders highlight critical policy areas to be addressed for positioning nursing colleges in higher education. These include gaps in educational resources, faculty qualifications, infrastructure, and research capacity. Staffing, infrastructure resources, and ongoing professional development are key policy pillars for organisational effectiveness. Strengthening the alignment between nursing education policies and the broader goals of higher education will not only sustain nursing colleges in meeting programme quality standards but also position them to contribute more effectively to the health workforce and health needs of populations.

## Data Availability

Datasets used and/or analysed during this study are available from the corresponding author on reasonable request.

## Abbreviations

CHE: Council on Higher Education
DHET: Department of Higher Education and Training
GANES: The Global Alliance for leadership in Nursing Education and Science
HEDIRCC: Higher Education Diagnostic Indicators of Readiness and Capacity to Change
IBM: International Business Machines
IQR: Interquartile Range
IT: Information Technology
KMO: Kaiser-Meyer-Olkin
OD: Organisational Development
PDoH: Provincial Department of Health
SPSS: Statistical Package for the Social Science
WHO: World Health Organisation
